# Supplementary material for: Low-Carbohydrate Diet among Children with Type 1 Diabetes: A Multi-Center Study
Source: Nutrients. 2021 Oct 30;13(11):3903. doi: 10.3390/nu13113903 (PMC8622801; doi:10.3390/nu13113903)
Supplement: Supplementary file 1 [file nutrients-13-03903-s001.zip › nutrients-1399866-supplementary.pdf]

Supplementary Table S1

Comparison of questionnaire respondents (N=624) to non-respondents (N=634). Data are shown as median (IQR).

	**Respondents**	**Non-respondents**	***p*-value**
**Subjects**	N = 624	N = 634	
**Sex**	F = 308 (49.4%)<br>M = 316 (50.6%)	F = 320 (50.5%)<br>M = 314 (49.5%)	0.69
**Age**	12.2 (6.2)	13.3 (6.4)	0.002**
**Age at T1D onset**	6.4 (6.2)	6.4 (6.2)	0.83
**Duration of T1D**	4.5 (5.3)	5.2 (6.3)	0.008**
**Treatment type**	MDI = 370 (59.4%)<br>CSII = 253 (40.6%)	MDI = 415 (65.5%)<br>CSII = 219 (34.5%)	0.03*
**HbA1c (mmol/mol)**	50.0 (14.0)	54.5 (16.0)	< 0.001***
**HbA1c (%)**	6.7 (3.4)	7.1 (3.6)	< 0.001***

CSII = continuous subcutaneous insulin infusion, F = female, M = male, MDI = multiple daily injection, T1D = type 1 diabetes

*p <0,05, **p <0,01, ***p <0,001

Supplementary Table S2

Comparison of subjects on very low-carbohydrate diet (N=5) vs. low-carbohydrate diet subjects (N=31). Data are shown as median (IQR).

	**Very low-carbohydrate diet**	**Low-carbohydrate diet**	***p*-value**
**Demographics**			
**Age at survey collection**	9.0 (5.8)	12.0 (5.7)	0.3
**Sex**	F = 2 (40%)<br>M = 3 (60%)	F = 23 (74.2%)<br>M = 8 (25.8%)	-
**Age at T1D onset (years)**	5.9 (5.7)	8.3 (7.4)	0.46
**T1D duration (years)**	3.2 (0.1)	3.2 (2.9)	0.75
**Anthropometric data**			
**Body height SDS**	-0.4 (0.9)	-0.3 (1.2)	1.00
**Body weight SDS**	1.8 (1.7)	0.4 (1.1)	0.22
**Body mass index SDS**	1.4 (1.0)	0.4 (1.1)	0.21
**Systolic arterial blood pressure (centile)**	81 (12.5)	38.5 (63.2)	0.08
**Diastolic arterial blood pressure (centile)**	72.5 (24.2)	59.5 (35.8)	0.22
**LCD data**			
**Daily carbohydrate intake**	35 (14.0)	100 (31)	< 0.001***
**Age at LCD start**	6.3 (5.6)	11.4 (6.1)	0.1
**LCD duration (years)**	3.0 (0.5)	0.9 (1.5)	0.004**
**Treatment and T1D control**			
**Treatment type**	MDI = 4 (80%)<br>CSII = 1 (20%)	MDI = 23 (74.1%)<br>CSII = 8 (25.8%)	-

**Bolus insulin (units daily)**	6.8 (4.1)	10.2 (9.2)	0.18
**Basal insulin (units daily)**	8.2 (5.6)	12.9 (10.6)	0.31
**Total insulin daily dose (units/kg/day)**	0.5 (0.1)	0.6 (0.3)	0.15
**Last HbA1c (mmol/mol)**	38.0 (10.0)	45.0 (9.5)	0.15
**Last HbA1c (%)**	5.6 (3.1)	6.3 (3.0)	0.15
**Average HbA1c during the last year (mmol/mol)**	43.0 (10.0)	49.0 (11.9)	0.16
**Average HbA1c during the last year (%)**	6.1 (3.1)	6.6 (3.2)	0.16
**CGM data**			
**Time in range 3.9-10.0 mmol/l (%)**	74.0 (1.1)	74.0 (16.1)	0.89
**Time below 3.9 mmol/l (%)**	15.0 (18.0)	6.0 (6.8)	0.12
**Time below 3.0 mmol/l (%)**	5.0 (6.2)	2.0 (3.0)	0.30
**Time above 10.0 mmol/l (%)**	2.0 (15.0)	17.0 (14.4)	0.12
**Time above 13.9 mmol/l (%)**	0.0 (2.0)	2.0 (3.0)	0.33
**Average glycemia (mmol/l)**	5.5 (1.6)	7.3 (1.1)	0.12
**Standard deviation of glycemia**	2.1 (1.1)	2.7 (0.9)	0.18
**Coefficient of variation (%)**	32.0 (10.0)	37.7 (7.2)	0.42
**Lipid spectrum**			
**Total cholesterol (mmol/l)**	5.4 (0.7)	4.7 (0.8)	0.05
**Triglycerides (mmol/l)**	1.4 (0.5)	0.9 (0.4)	0.10
**HDL cholesterol (mmol/l)**	1.3 (0.2)	1.5 (0.5)	0.08
**LDL cholesterol (mmol/)**	3.2 (0.8)	2.6 (1.0)	0.57

CGM = continuous glucose monitoring, CSII = continuous subcutaneous insulin infusion, F = female, IQR = interquartile ratio, HDL = high density lipoprotein, LCD = low-carbohydrate diet, LDL = low density lipoprotein, M = male, MDI = multiple daily injection, SDS = standard deviation score, T1D = type 1 diabetes, VLCD = very low-carbohydrate diet

*p <0,05, **p <0,01, ***p <0,001
